# Mood Disorders and Dysautonomia in Patients Diagnosed with Idiopathic Hypersomnia: A Retrospective Analysis (2000–2023)

**DOI:** 10.3390/jcm14134593

**Published:** 2025-06-28

**Authors:** Roger Rochart, Rena Y. Jiang, Irene Chu, Hope Kincaid, Martina Vendrame

**Affiliations:** 1 Lehigh Valley Fleming Neuroscience Institute, Lehigh Valley Health Network, Allentown, PA 18104, USA; rogerrochart@gmail.com (R.R.); jiang.rena.030@gmail.com (R.Y.J.); irene.chu@jefferson.edu (I.C.); 2 Network Office of Research and Innovation, Lehigh Valley Health Network, Allentown, PA 18104, USA; hope.kincaid@jefferson.edu

**Keywords:** idiopathic hypersomnia, Epworth Sleepiness Scale, polysomnography, multiple sleep latency test, sleep inertia, Sleep Onset REM Period, depression, anxiety, dysautonomia, postural orthostatic tachycardia syndrome

## Abstract

**Background/Objectives:** There is limited data on well-documented comorbidities with polysomnography (PSG)/multiple sleep latency test (MSLT) findings in idiopathic hypersomnia (IH). We aimed to characterize the clinical, PSG/MSLT characteristics of IH patients in our health network. **Methods**: We reviewed charts of all IH cases between 2000 and 2023, extracting clinical features, comorbidities, and PSG/MSLT findings. **Results:** One hundred forty-two patients (83.80% female) with IH were included. Compared to those without mood disorders, both major depressive disorder (MDD) and anxiety patients were older at onset (27.10 ± 8.32 and 26.76 ± 8.40 versus 23.23 ± 6.94 and 24.05 ± 7.31 years; *p* = 0.003 and *p* = 0.042) and had lower ESS (15 versus 19; 15.67 versus 17.75; *p* < 0.0001), more disrupted sleep (28 (36.36%) versus 8 (12.31%); *p* = 0.001; 24 (35.82%) versus 12 (16%); *p* = 0.007), and less sleep inertia (30 (38.96%) versus 38 (58.46%); *p* = 0.021; 26 (38.81%) versus 42 (56%); *p* = 0.04). Fifteen patients with dysautonomia disorders presented at an earlier age (21.80 ± 6.60 versus 25.75 ± 8, *p* = 0.0682). On MSLT, MDD, anxiety, and dysautonomia patients had longer sleep latencies than the non-affected counterparts (6.40 (5.40–7.60) minutes versus 3.60 (2.60–5.40) min., <0.0001; 6.20 (5.20–7.40) versus 4 (2.60–6.40) minutes; *p* < 0.0001; 7.40 (6–7.80) versus 5.40 (3–7); *p* = 0.008). MDD and anxiety cases had fewer sleep onset REM periods (7 (9.09%) versus 16 (24.62%), *p* = 0.0124 and 6 (8.96%) versus 17 (22.67%), *p* = 0.0388) compared to those not affected by these disorders. **Conclusions:** Our study highlights the importance of recognizing mood disorders and dysautonomia in patients diagnosed with IH. Further research may elucidate management strategies for these patients.

## 1. Introduction

Idiopathic hypersomnia (IH) is an uncommon and chronic sleep disorder initially described by Bedrich Roth in Prague in 1956 [1]. This condition is characterized by excessive daytime sleepiness, an irresistible urge to sleep accompanied by prolonged, non-refreshing naps, and difficulty waking up from sleep despite experiencing normal or extended nocturnal sleep [1].

Since its initial identification, there has been an ongoing evolution in the nomenclature, diagnostic approach, symptom characterization, and understanding of the disease burden. Increased attention has been directed towards studying epidemiology, neurobiology, and potential therapeutic strategies, contributing to an enhanced understanding for identification and treatment [2,3]. Currently, idiopathic hypersomnia is classified as an orphan disease with an unknown frequency, and its cause remains elusive. Nevertheless, evidence suggests that circadian and sleep structure variances, structural brain changes, and neurochemical alterations may play a role in the development and manifestation of this disorder.

The diagnosis of IH relies on objective sleep testing with overnight polysomnography (PSG) and a multiple sleep latency test (MSLT), in addition to the presence of associated clinical features [4,5]. However, clinicians may face challenges in recognizing and accurately diagnosing it due to its low prevalence, clinical diversity, and symptom overlap with other sleep disorders [4]. The Lehigh Valley Health Network (LVHN) sleep center is a tertiary sleep disorder referral center serving a population of about 3.3 million. This study’s purpose is to describe the prevalence and clinical and polysomnographic characteristics of IH, with a focus on comorbidities, based on a large cohort of patients.

## 2. Materials and Methods

Internal Institutional Review Board (IRB) approval was obtained before initiating the study. All study data was recorded and maintained in Research Electronic Data Capture (REDCap), a secure, web-based software platform designed to support data capture for research studies [6,7].

We undertook a retrospective analysis of all adult patients (18 years or older) with IH seen at LVHN between 1 January 2000 and 31 December 2023. The system database was queried for “Idiopathic Hypersomnia”. All charts of patients with a diagnosis of IH based on the International Classification of Sleep Disorders-3 (ICSD-3) diagnostic criteria for IH were included [5]. All charts were reviewed by a board-certified sleep specialist (MV) to confirm accurate diagnosis.

Data on demographics, IH-specific clinical information (age at disease onset, age at diagnosis, presence and severity of daytime sleepiness with the Epworth Sleepiness Scale score (ESS)), cataplexy, sleep paralysis, or hypnagogic hallucinations, presence of disrupted sleep, need for restorative naps, non-refreshing naps, information on IH workup with overnight PSG and MSLT, sleep comorbidities, other comorbidities including neurological disorders, dysautonomia disorders (neurocardiogenic syncope, postural orthostatic tachycardia syndrome (POTS)), psychiatric disorders (anxiety disorder, MDD, attention deficit disorder, obsessive compulsive disorder, post-traumatic stress disorder, psychological non-epileptic seizures, other conversion disorders, psychosis/schizophrenia), cardiovascular disorders, rheumatic disorders, gastrointestinal disorders, metabolic disorders, endocrine disorders, and chronic pain disorders were collected. The diagnosis of neurocardiogenic syncope and POTS were made by cardiology after appropriate testing (including tilt-table test). Psychiatry diagnoses were made by qualified psychiatrists using validated standardized assessment tools.

PSG and MSLT of all patients were reviewed and all recorded parameters were collected. On the MSLT, we collected the number of sleep onset REM periods (SOREMPs), the sleep time in each nap, and average sleep latency on MSLT. MSLTs were conducted following AASM guidelines [8]. Urine drug tests were obtained prior to testing and medications with alerting, sedating, and/or REM-sleep-modulating properties were tapered down at least 2 weeks before the MSLT [8].

### Statistical Analysis

Continuous variables are reported as the mean and standard deviation (SD) or the median and interquartile range (IQR), depending on normality. Skewness was assessed through visual inspection of histograms and the skewness statistic. A skewness statistic less than −1 or greater than +1 is considered skewed. Frequencies and percentages are reported for categorical variables. Bivariate analyses were conducted to determine if IH-specific features such as severity of sleepiness, PSG/MSLT features, presenting symptoms, and response to treatment are associated with type of comorbid disorder. The chi-square or Fisher’s exact test was used to compare categorical variables between groups. The independent samples *t*-test or Mann–Whitney U test was used to compare continuous variables. All analyses were two-tailed, and alpha was set at 0.05. Statistical analysis was performed using SAS 9.4 (SAS Institute Inc., Cary, NC, USA).

## 3. Results

### 3.1. Clinical, PSG, and MSLT Characteristics of the Whole Cohort 

We included 142 patients diagnosed with IH, based on the ICSD-3 criteria. Age at onset was 25.33 ± 7.93 years and 119 (83.80%) were female. Average ESS was 16.77 ± 2.92. Demographics and clinical characteristics of the whole cohort are presented in Table 1. The most common presenting symptoms were sleep inertia (68 (47.89%)), non-refreshing naps (48 (33.80%)), and disrupted sleep (36 (25.35%)).

Review of overnight PSG data of all patients showed a median sleep efficiency of 90% (IQR:86–95%), sleep latency of 13.50 min (IQR:6–33), and REM latency of 122.50 min (IQR:79.50–183.50). On MSLT, average sleep latency was 5.21 ± 2.08 min. Only 23 (16.20%) patients had sleep onset REM periods (SOREMP) on MSLT.

Table 2 introduces the comorbidities within the whole cohort. MDD and anxiety were the most common comorbidities, diagnosed in 54% and 47% of patients, respectively. Other common comorbidities were primary headaches (34%), chronic pain (27%), irritable bowel syndrome (14%), fibromyalgia (12%), and dysautonomia disorders (11%).

### 3.2. Clinical and Polysomnographic Features of Patients with Comorbid Mood Disorders

**Figure 1:**
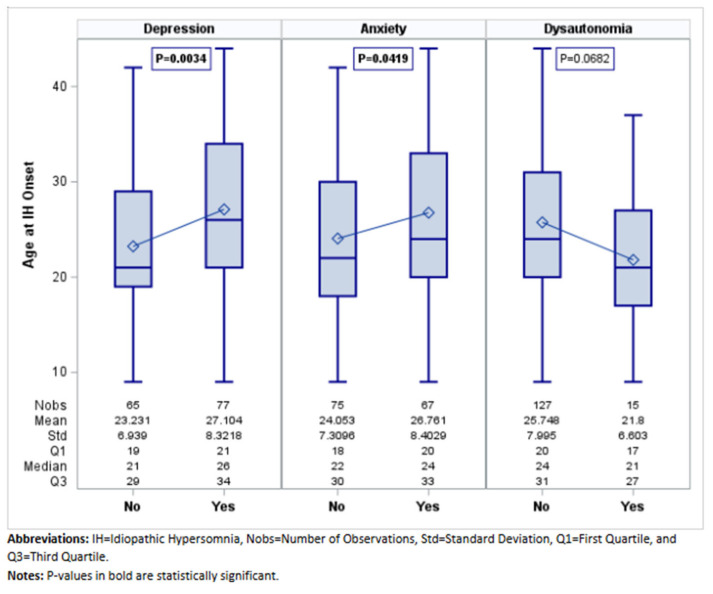
Age at IH Onset by Comorbid Condition.

**Figure 2:**
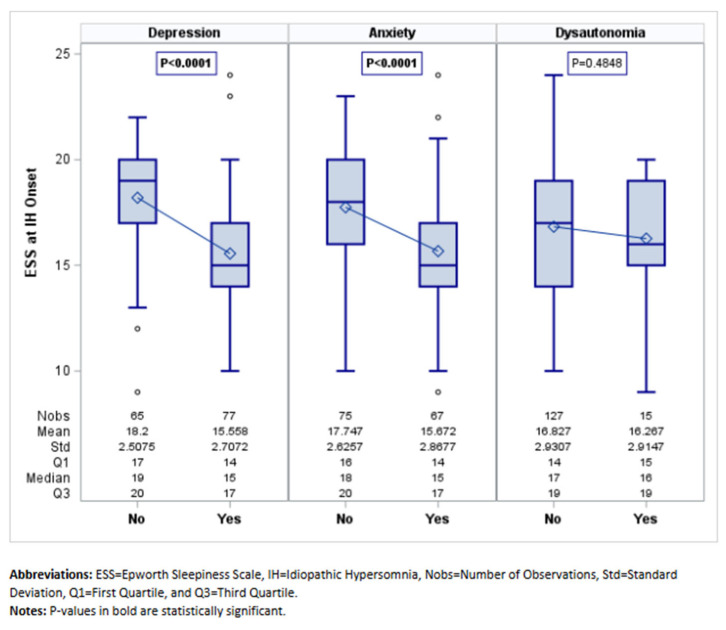
ESS at IH Onset by Comorbid Condition.

**Figure 3:**
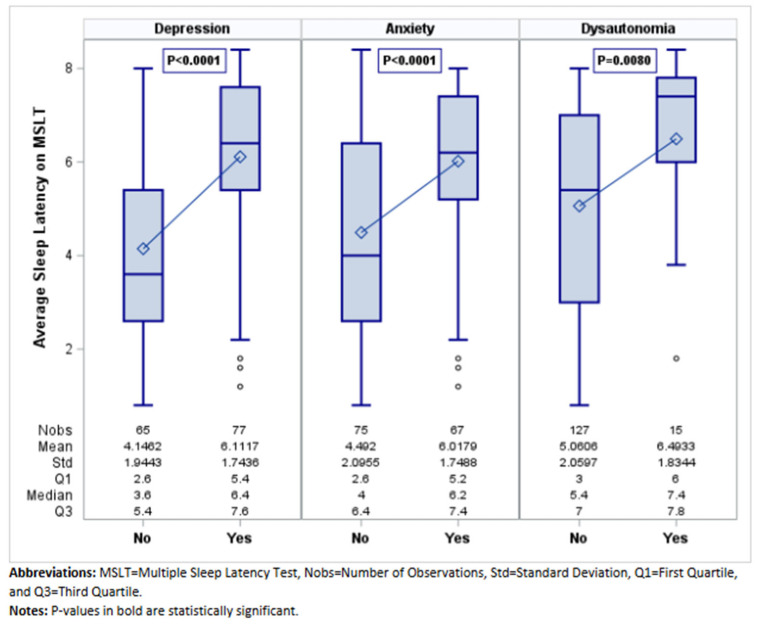
Average Sleep Latency on MSLT by Comorbid Condition.

MDD was the most frequent mood disorder (77 (54.23%)) (Table 3). MDD symptoms were present before the diagnosis of IH in most cases (63, 82%). Compared to patients without MDD, MDD patients had older age at onset (27.10 ± 8.32 versus 23.23 ± 6.94 years; *p* = 0.003) (Figure 1) and lower ESS (15 versus 19; *p* < 0.0001) (Figure 2), reported more frequently disrupted sleep (28 (36.36%) versus 8 (12.31%); 0.001), and had less non-refreshing naps (16 (20.78%) versus 32 (49.23%); *p* < 0.001) and less sleep inertia (30 (38.96%)) versus 38 (58.46%); *p* = 0.021). On PSG, MDD patients had longer sleep latency (15.50 (7–36.50) versus 9.50 (4–22.50) minutes; *p* = 0.002). On MSLT, patients with MDD had a longer sleep latency ((6.40 (5.40–7.60) versus 3.60 (2.60–5.40); *p* < 0.001) (Figure 3), and fewer patients with MDD (7 (9.09%)) had SOREMPs compared to patients without MDD (16 (24.62%)) (*p* = 0.0124) (Table 3).

Sixty-seven (47.18%) patients had a diagnosis of anxiety disorder (Table 4). Anxiety symptoms preceded IH diagnosis in most cases (51 of the 67 cases, 76%). Compared to patients without anxiety, anxiety patients had older age at onset (26.76 ± 8.40 versus 24.05 ± 7.31 years; *p* = 0.042) (Figure 1), lower ESS (15.67 ± 2.87 versus 17.75 ± 2.63; *p* < 0.001) (Figure 2), and fewer episodes of abruptly falling asleep (3 (4.48%) versus 12 (16%); *p* = 0.026). Similar to MDD patients, they reported more frequently disrupted sleep (24 (35.82%) versus 12 (16%); *p* = 0.007) and less sleep inertia (26 (38.81%) versus 42 (56%); *p* = 0.041). On PSG, anxiety patients had lower sleep efficiency (88% versus 92%; *p* = 0.01) (Table 4). On MSLT, patients with anxiety had longer sleep latencies (6.20 (5.20–7.40) minutes versus 4 (2.60–6.40), <0.0001) (Figure 3). Fewer patients with anxiety (6 (8.96%)) had SOREMPs on MSLT compared to patients without anxiety (17 (22.67%)) (*p* = 0.039) (Table 4).

### 3.3. Clinical and Polysomnographic Features of Patients with Dysautonomia Disorders

Among the whole cohort we identified 15 patients (10.56%) with dysautonomia disorders, 8 (5.63%) with a diagnosis of recurrent vasovagal syncope, and 12 (8.45%) with a diagnosis of POTS after cardiovascular work up. Clinical and polysomnographic features of patients with and without dysautonomia are presented in Table 5 Patients with dysautonomia were younger, at symptom onset, than those without dysautonomia (21.80 ± 6.60 versus 25.75 ± 8; *p* = 0.068) (Figure 1). However, they presented with a similar degree of sleepiness (ESS 16.27 ± 2.91) (Figure 2) and similar presenting symptoms than the other patients without dysautonomia. Analysis of MSLT features showed a longer sleep latency on MSLT for patients with dysautonomia (7.40 (6–7.80) versus 5.40 (3–7); *p* = 0.008) (Table 5; Figure 3).

## 4. Discussion

This study is one of the largest monocentric studies to date of patients with IH as diagnosed based on ICSD-3 criteria. We identified specific clinical and polysomnographic characteristics of patients diagnosed with IH who had also been diagnosed with MDD, anxiety, or dysautonomia disorders. Both patients with MDD and anxiety presented at older age, were less sleepy, and reported more frequently disrupted sleep than their non-affected counterparts. Both were found to have less “sleep inertia”. MDD patients also reported non-refreshing naps less frequently than other IH patients without MDD. Distinctly, patients with anxiety reported episodes of “abruptly falling asleep” less frequently than IH patients without anxiety.

Subjective symptoms reported by patients with IH overlap with symptoms of depression [9,10]. Patients with IH often present with pronounced symptoms of sleep inertia and non-refreshing naps [11]. Compared to patients with narcolepsy, these features are recognized as more characteristic of IH. Sleep inertia, defined as “severe difficulty transitioning from sleep to wakefulness”, is a hallmark symptom that can significantly impair daily functioning. Similarly, naps in IH patients tend to be prolonged and unrefreshing, contrasting with the typically refreshing naps reported by narcolepsy patients [11,12]. The persistence of unrefreshing naps despite extended total sleep time in IH suggests that the quality of sleep is fundamentally altered, contributing to the profound daytime sleepiness experienced by these patients [13]. Although patients with depression may also report “sleep inertia” and “non-refreshing naps”, we observed that these symptoms were not as often as reported by patients with IH only. It is important to note our definition of both these symptoms. While patients may experience “difficulty getting out of bed in the morning”, we considered “sleep inertia” only a significant difficulty with wakening, requiring multiple alarm clocks usually set at a distance or secondary help with wakefulness. Similarly, because often patients may not necessarily feel great after a nap, we considered “non-refreshing naps” only “naps followed by difficulty waking and or worsening sleepiness than before initiating sleep”.

Analysis of polysomnographic data showed findings aligning with current evidence on nocturnal sleep architecture in IH [14]. Comparing polysomnographic data of patients with MDD or anxiety to those without, we found that both MDD and anxiety were associated with longer sleep latencies and most commonly no SOREMPs on MSLT. Furthermore, both subsets showed lower sleep efficiency than the non-affected counterparts, although statistical significance was reached only for the anxiety group. Previous studies have highlighted that individuals with depression or anxiety often exhibit specific polysomnographic changes, including prolonged sleep onset latency, reduced REM latency, and increased REM sleep density [15]. In patients with IH, depression has been shown to be associated with poor sleep efficiency, further differentiating this population from those with IH alone [10]. These findings suggest that mood disorders may amplify the existing dysregulation of sleep architecture seen in IH. The shared neurobiological pathways between hypersomnolence and mood disorders, such as dysregulation of the hypothalamic–pituitary–adrenal axis and abnormalities in serotoninergic and GABAergic signaling, may underlie these observed differences [16,17]. Further research should explore these mechanisms to clarify how mood disorders contribute to the pathophysiology of hypersomnia.

Our findings may also suggest that IH patients with depression or anxiety represent a distinct subset of IH patients, characterized by more pronounced symptoms of mood disorders and less prominent IH-specific features, such as severe daytime sleepiness, sleep inertia, and unrefreshing naps. A notable observation is that, in most patients with both IH and mood disorders, the symptoms of MDD or anxiety preceded the diagnosis of IH. This temporal pattern may suggest that the symptoms of mood disorders may be not only closely linked to IH but could also lead to the development of hypersomnia. However, at the time of assessment, mood disorder symptoms were not considered the primary cause of hypersomnia, leading to the diagnosis of IH. These observations call into question whether the hypersomnia diagnosed in this subgroup of patients was truly idiopathic or, instead, secondary to underlying depressive pathology.

Hypersomnia is recognized as a symptom of MDD, but there is limited evidence about the longitudinal association between hypersomnia and MDD, as well as the potential causal role of mood disorders in the development of hypersomnolence. The study by Plante et al. showed that the odds for development of depression were significantly increased 1.67-fold in participants who also reported subjective daytime sleepiness [14,18]. Interestingly, a reduced mean sleep latency <8 min on the MSLT was associated with a trend towards reduced odds of developing depression, suggesting that increased physiological sleep propensity, as observed in patients with IH, was associated with a decreased likelihood of depression onset [18]. These findings highlight the complexity of the relationship between hypersomnolence and depression, suggesting that subjective sleepiness and objective sleep propensity may have divergent implications for mood disorder risk. Further longitudinal research is needed to clarify these associations and to better understand the underlying mechanisms linking hypersomnia and MDD.

Among the whole cohort we identified 15 patients with dysautonomia disorders who were younger than the other patients in the cohort. Those patients were diagnosed with IH at a younger age, but they presented with a similar degree of sleepiness and presenting symptoms to the other patients without dysautonomia. Similarly to what was found for cases of depression and anxiety, analysis of PSG and MSLT features showed a longer sleep latency on MSLT for patients with dysautonomia. Dysautonomia disorders in patients with IH have been reported previously [19,20,21]. The observed autonomic symptoms suggest a potential pathophysiological link between autonomic nervous system (ANS) dysfunction and hypersomnolence. Our findings support the growing body of evidence indicating that autonomic symptoms, such as orthostatic intolerance, vasomotor instability, and gastrointestinal disturbances, are prevalent in IH [21]. These symptoms are not only more frequent but also more severe in IH patients compared to healthy controls, suggesting systemic dysregulation that extends beyond hypersomnolence. The link between ANS dysfunction and hypersomnolence can be partially explained by the role of the ANS in regulating sleep and wakefulness [21]. Dysregulation of the parasympathetic and sympathetic branches may disrupt normal arousal mechanisms, leading to excessive daytime sleepiness and unrefreshing sleep. For instance, increased parasympathetic tone, which has been observed in IH patients, may contribute to prolonged sleep inertia and difficulty with awakening. Conversely, impaired sympathetic activation may result in reduced alertness and heightened fatigue during waking hours. Our initial results further highlight the interplay between ANS dysfunction and hypersomnolence. Patients with higher autonomic symptom burden reported more severe sleep inertia and fatigue. These findings align with previous research suggesting that autonomic symptoms are positively correlated with measures of excessive daytime sleepiness and negatively correlated with quality of life [21]. Lastly, the presence of mood disorders in IH patients may exacerbate ANS dysfunction, creating a feedback loop that amplifies hypersomnolence.

We observed that mood disorders, dysautonomia, and hypersomnia frequently coexist and exhibit complex bidirectional interactions that complicate diagnosis and management. Mood disorders such as depression and anxiety may exacerbate autonomic dysfunction, contributing to symptoms like orthostatic intolerance, tachycardia, and fatigue, which are hallmarks of ANS dysfunction. In turn, dysautonomia-related disruptions in cardiovascular and thermoregulatory homeostasis may impair sleep architecture and exacerbate mood instability. Hypersomnia, whether idiopathic or secondary, often overlaps with both conditions, presenting as excessive daytime sleepiness that may stem from or contribute to ANS dysregulation and mood disturbance. Clinically, this triad may result in non-restorative sleep, heightened fatigue, and impaired functional capacity. We have observed polysomnographic findings of reduced sleep efficiency, increased sleep latency, or fragmented sleep. Others have shown that these markers may not always correlate with subjective symptom severity [14].

Comprehensive management of these patients requires an integrative approach addressing neuropsychiatric, autonomic, and sleep-related components concurrently. Psychiatric evaluation and treatment, including pharmacologic and psychotherapeutic interventions, are essential to stabilize mood and reduce cognitive–emotional contributors to autonomic arousal. Concurrently, autonomic symptoms must be addressed through tailored strategies (such as volume expansion, graded exercise) and medications like beta-blockers or fludrocortisone, depending on the specific dysautonomic profile (e.g., POTS or neurocardiogenic syncope). In parallel, hypersomnia should be evaluated through polysomnography and MSLT, with treatment options ranging from sleep hygiene to wake-promoting agents such as modafinil or pitolisant.

Several limitations should be acknowledged. The retrospective nature of the study introduces inherent biases, as data were gathered from patient-reported symptoms during their initial encounter at the sleep clinic. For instance, some patients may not have reported “non-refreshing naps” even though they were not experiencing “refreshing naps”. This reliance on self-reported data increases the potential for recall bias and variability in symptom reporting.

Our study further limits causal inferences regarding the relationships between comorbidities (such as depression and anxiety), autonomic dysfunction, and IH symptoms. Longitudinal data from prospective studies will be necessary to determine the temporal sequence of these associations. Furthermore, the small sample size in certain subgroups, such as patients with specific comorbidities, reduces the statistical power to detect meaningful differences or trends. Finally, the study’s reliance on data collected from a specialized sleep clinic within LVHN only may introduce population and referral bias. The LVHN encompasses a specific geographic region within Pennsylvania, limiting the population that may present with symptoms of IH and reducing the generalizability and power of our findings.

## 5. Conclusions

Our study highlights significant clinical and polysomnographic differences in IH patients with and without MDD, anxiety, or dysautonomia disorders. These findings underscore the importance of recognizing these comorbidities in IH, as it may influence clinical presentation and diagnostic features. Addressing these symptoms requires a tailored approach that considers the distinct clinical features of IH compared to other hypersomnolence disorders. This study also emphasizes the role of mood disorders, such as depression, anxiety, and dysautonomia, in shaping the clinical presentation of IH. Comprehensive management of patients with co-occurring mood disorders, dysautonomia, and hypersomnia necessitates an integrative, multidisciplinary approach that simultaneously targets neuropsychiatric, autonomic, and sleep-related dysfunctions. Coordination across neurology, psychiatry, sleep medicine, and, in some cases, cardiology or autonomic specialists is vital, as isolated treatment of one domain often yields limited benefit or exacerbates symptoms in others. Optimal outcomes are more likely when management is dynamic, individualized, and informed by ongoing reassessment of symptom interplay and treatment response. Further prospective research will elucidate underlying mechanisms and optimize management strategies for these patients.

## Figures and Tables

**Table 1:** Demographics and clinical characteristics of the whole cohort.

Variable	Entire Sample (N = 142)
Current Age *, yrs mean ± SD	38.54 ± 10.68
Age at onset, yrs mean ± SD	25.33 ± 7.93
Age at onset (Cat.) n (%)	
0–18	27 (19.01)
19–29	71 (50)
30–39	34 (23.94)
40–49	10 (7.04)
Age at diagnosis, yrs mean ± SD	32.37 ± 9.81
Age at diagnosis (Cat.) n (%)	
0–18	8 (5.63)
19–29	55 (38.73)
30–39	44 (30.99)
40–49	30 (21.13)
50–59	5 (3.52)
Time onset to diag, yrs median(IQR)	4.80 (1.50–10.90)
Gender n (%)	
Male	23 (16.20)
Female	119 (83.80)
Race n (%)	
White/Caucasian	121 (85.21)
Black or African American	4 (2.82)
Asian	3 (2.11)
American Indian or Alaska Native	-
Multi-racial	6 (4.23)
Other	5 (3.52)
Unknown: Patient unsure of race	1 (0.70)
Unknown/Missing	2 (1.41)
IH Symptoms	
Excessive daytime sleepiness	142 (100)
Abruptly falling asleep	15 (10.56)
Cataplexy	1 (0.70)
Sleep paralysis	8 (5.63)
Hypnagogic hallucinations	10 (7.04)
Hypnopompic hallucinations	1 (0.70)
Difficulty falling asleep at night	15 (10.56)
Disrupted sleep	36 (25.35)
Refreshing naps	8 (5.63)
Non-refreshing naps	48 (33.80)
Sleep inertia	68 (47.89)
Other	1 (0.70)
ESS at onset (0–24) median (IQR)	17 (15–19)
ESS on treatment or last appt (0–24) mean ± SD	11.21 ± 4.66

Abbreviations: IH = Idiopathic Hypersomnia, yrs = Years, SD = standard deviation, Cat = Categorical, Diag = Diagnosis, IQR = interquartile range, and ESS = Epworth Sleepiness Scale. Notes: * Current age calculated as of 12/13/24. Categorical variables are presented as the frequency (n) and percentage (%). Percentages are based upon column totals, unless otherwise noted next to the variable name. Continuous variables are presented as the mean ± SD if normally distributed or median and IQR if not.

**Table 2:** Comorbidities within the whole cohort.

Comorbid Condition	All Patients (N = 142)
Neurological Disorders n (%)	68 (47.89)
TBI	8 (5.63)
TIA/Stroke	2 (1.41)
Parkinson Disease	0
Primary Headaches	49 (34.51)
Secondary Headaches	9 (6.34)
Facial Pain/Trigeminal Neuralgia	7 (4.93)
Seizures/Epilepsy	1 (0.70)
Brain Tumor	1 (0.70)
MS/Demyelinating Disease	0
Other	6 (4.23)
Unknown/Missing	0
Sleep Disorder n (%)	42 (29.58)
Insomnia	7 (4.93)
OSA	18 (12.68)
CSA	0
Non-REM Parasomnias	1 (0.70)
PLMD	4 (2.82)
RLS	14 (9.86)
REM Parasomnia	1 (0.70)
Circadian Rhythm Sleep–Wake Disorders	2 (1.41)
Other	0
Unknown/Missing	0
Psychiatric Disorders n (%)	93 (65.49)
Anxiety	67 (47.18)
Depression	77 (54.23)
Bipolar Disorder	11 (7.75)
Schizophrenia	0
Intellectual Disability	1 (0.70)
ADHD/ADD	16 (11.27)
OCD	2 (1.41)
PTSD	26 (18.31)
Psychogenic Non-epileptic Seizures	2 (1.41)
Other Conversion Disorders	2 (1.41)
Disruptive Behavior and Dissocial Disorders	0
ASD/Communications Disorders	0
Eating Disorders	1 (0.70)
Other	8 (5.63)
Dysautonomia n (%)	15 (10.56)
Vasovagal Syncope	8 (5.63)
POTS	12 (8.45)
Other	0
Cardiovascular Disorders n (%)	21 (14.79)
HTN	16 (11.27)
CHF/Cardiomyopathy	1 (0.70)
Arrhythmia	3 (2.11)
Other	3 (2.11)
Metabolic Disorders n (%)	57 (40.14)
Diabetes	9 (6.34)
Obesity	42 (29.58)
Hyperlipidemia	30 (21.13)
Other	0
Rheumatic Disorders n (%)	23 (16.20)
Rheumatoid Arthritis	0
Lupus	0
Osetoarthritis	1 (0.70)
Ehlers–Danlos Syndrome	2 (1.41)
Fibromyaglia	17 (11.97)
Other	6 (4.23)
Gastrointestinal Disorders n (%)	34 (23.94)
Crohn’s Disease/Ulcerative Colitis	3 (2.11)
Celiac Disease	4 (2.82)
IBS	20 (14.08)
Other	14 (9.86)
Endocrine Disorders n (%)	25 (17.61)
Hyperthyroidism	2 (1.41)
Hypothyroidism	22 (15.49)
Pituitary Disorders	0
Adrenal Insufficiency	0
Other	2 (1.41)
Chronic Pain Disorder n (%)	38 (26.76)
Chronic Neck Pain	13 (9.15)
Chronic Back Pain	29 (20.42)
Other	4 (2.82)

Abbreviations: TBI = Traumatic Brain Injury, TIA = Transient Ischemic Attack, MS = Multiple Sclerosis, OSA = Obstructive Sleep Apnea, CSA = Central Sleep Apnea, REM = Rapid Eye Movement, PLMD = Periodic Limb Movement Disorder, RLS = Restless Leg Syndrome, ADHD = Attnetion Deficit Hyperactivity Disorder, ADD = Attention Deficit Disorder, OCD = Obsessive Compulsive Disorder, PTSD = Post-traumatic Stress Disorder, ASD = Autism Spectrum Disorder, POTS= Postural orthostatic tachycardia syndrome, HTN = Hypertension, CHF = Congestive Heart Failure, and IBS = Irritable Bowel Syndrome. Notes: Categorical variables are presented as the frequency (n) and percentage (%). Percentages are based upon column totals, unless otherwise noted next to the variable name.

**Table 3:** Clinical and polysomnographic features of patients with and without comorbid depression.

**(a) Clinical Features**			
**Variable**	**Depression** **(n = 77)**	**No Depression** **(n = 65)**	***p*-Value**
IH Symptoms			
Excessive daytime sleepiness	77 (100)	65 (100)	NA
Abruptly falling asleep	5 (6.49)	10 (15.38)	0.0859 ^b^
Cataplexy	0	1 (1.54)	0.4577 ^c^
Sleep paralysis	4 (5.19)	4 (6.15)	1.0000 ^c^
Hypnagogic hallucinations	4 (5.19)	6 (9.23)	0.5126 ^c^
Hypnopompic hallucinations	1 (1.30)	0	1.0000 ^c^
Difficulty falling asleep at night	10 (12.99)	5 (7.69)	0.3065 ^a^
Disrupted sleep	28 (36.36)	8 (12.31)	**0.0010 ^b^**
Refreshing naps	4 (5.19)	4 (6.15)	1.0000 ^c^
Non-refreshing naps	16 (20.78)	32 (49.23)	**0.0004 ^b^**
Sleep inertia	30 (38.96)	38 (58.46)	**0.0205 ^b^**
Other	1 (1.30)	0	1.0000 ^c^
ESS at onset (0–24) median (IQR)	15 (14–17)	19 (17–20)	**<0.0001 ^d^**
ESS after tx or last appt (0–24) mean ± SD	11.36 ± 4.91	11.03 ± 4.37	0.6727 ^a^
Abbreviations: IH = Idiopathic Hypersomnia, ESS = Epworh Sleepiness Scale, IQR = Interquartile Range, tx = Treatment, appt = Appointment, and SD = Standard Deviation. Notes: Categorical variables are presented as the frequency (n) and percentage (%). Percentages are based upon column totals, unless otherwise noted next to the variable name. Continuous variables are presented as the mean ± SD if normally distributed or median and IQR if not. Statistically significant *p*-values that are in bold. a. *p*-value generated using the independent Samples *t*-test. b. *p*-value generated using the chi-square test of Independence. c. *p*-value generated using Fisher’s exact test. d. *p*-value generated using the Mann-Whitney U test.			
**(b) Polysomnography Features**			
**Variable**	**Depression** **(n = 84)**	**No Depression** **(n = 72)**	***p*-Value**
Total sleep time, min mean ± SD	399.73 ± 31.86	408.13 ± 32.71	0.1071 ^a^
Total sleep efficiency, % median (IQR)	88.50 (86–94)	92 (87–95)	0.1101 ^b^
Total sleep latency, min median (IQR)	15.50 (7–36.50)	9.50 (4–22.50)	**0.0024 ^b^**
Total REM latency, min median (IQR)	131.50 (79.50–185.50)	111 (79.50–178.50)	0.4196 ^b^
Abbreviations: min = minutes, SD = Standard Deviation, IQR = Interquartile Range, and REM = Rapid Eye Movement. Notes: Continuous variables are presented as the mean ± SD if normally distributed, median and IQR if not. Statistically significant *p*-values are in bold. a. *p*-values generated using the independent samples *t*-test. b. *p*-value generated using the Mann-Whitney U test.			
**(c) Multiple Sleep Latency Test (MSLT) Features**			
**Variable**	**Depression** **(n = 77)**	**No Depression** **(n = 65)**	***p*-Value**
Avg sleep latency, min median (IQR)	6.40 (5.40–7.60)	3.60 (2.60–5.40)	**<0.0001 ^a^**
Avg SOREMPs, min mean ± SD (n = 23)	8.71 ± 4.68	9.19 ± 4.65	0.8248 ^b^
Num naps w/ SOREMPS n (%)			**0.0124 ^c^**
0	70 (90.91)	49 (75.38)	
1	7 (9.09)	16 (24.62)	
Abbreviations: MSLT = Multiple Sleep Latency Test, Avg = Average, Min = Minutes, IQR = interquartile range, SOREMPS = Sleep Onset Rapid Eye Movement Periods, SD = Standard Deviation, Num = Number, w/ = With. Notes: Categorical variables are presented as the frequency (n) and percentage (%). Percentages are based upon column totals, unless otherwise noted next to the variable name. Continuous variables are presented as the mean ± SD if normally distributed, median and IQR if not. Statistically significant *p*-values are in bold. a. *p*-value generated using the Mann-Whitney U test. b. *p*-value generated using the independent samples *t*-test. c. *p*-value generated using the chi-square test of independence.			

**Table 4:** Clinical and polysomnographic features of patients with and without comorbid anxiety.

**(a) Clinical Features**			
**Variable**	**Anxiety (n = 67)**	**No Anxiety (n = 75)**	***p*-Value**
IH Symptoms			
Excessive daytime sleepiness	67 (100)	75 (100)	NA
Abruptly falling asleep	3 (4.48)	12 (16)	**0.0257 ^b^**
Cataplexy	1 (1.49)	0	0.4718 ^c^
Sleep paralysis	4 (5.97)	4 (5.33)	1.0000 ^c^
Hypnagogic hallucinations	7 (10.45)	3 (4)	0.1909 ^c^
Hypnopompic hallucinations	0	1 (1.33)	1.0000 ^c^
Difficulty falling asleep at night	10 (14.93)	5 (6.67)	0.1100 ^b^
Disrupted sleep	24 (35.82)	12 (16)	**0.0067 ^b^**
Refreshing naps	5 (7.46)	3 (4)	0.4758 ^c^
Non-refreshing naps	18 (26.87)	30 (40)	0.0986 ^b^
Sleep inertia	26 (38.81)	42 (56)	**0.0406 ^b^**
Other	0	1 (1.33)	1.0000 ^c^
ESS at onset (0–24) mean ± SD	15.67 ± 2.87	17.75 ± 2.63	**<0.0001 ^a^**
ESS after tx or last appt (0–24) mean ± SD	10.96 ± 4.43	11.44 ± 4.87	0.5375 ^a^
Abbreviations: IH = Idiopathic Hypersomnia, ESS = Epworh Sleepiness Scale, SD = Standard Deviation, tx = Treatment, and appt = Appointment. Notes: Categorical variables are presented as the frequency (n) and percentage (%). Percentages are based upon column totals, unless otherwise noted next to the variable name. Continuous variables are presented as the mean ± SD if normally distributed or median and IQR if not. Statistically significant *p*-values that are in bold. a. *p*-value generated using the independent Samples *t*-test. b. *p*-value generated using the chi-square test of Independence. c. *p*-value generated using Fisher’s exact test.			
**(b) Polysomnography Features**			
**Variable**	**Anxiety (n = 73)**	**No Anxiety (n = 83)**	***p*-Value**
Total sleep time, min mean ± SD	398.71 ± 34.62	407.91 ± 29.91	0.0767 ^a^
Total sleep efficiency, % median (IQR)	88 (86–94)	92 (87–95)	**0.0099 ^b^**
Total sleep latency, min median (IQR)	17 (7–34)	10 (5–26)	0.0547 ^b^
Total REM latency, min median (IQR)	134 (85–186)	108 (79–177)	0.3199 ^b^
Abbreviations: min = minutes, SD = Standard Deviation, IQR = Interquartile Range, and REM = Rapid Eye Movement. Notes: Continuous variables are presented as the mean ± SD if normally distributed, median and IQR if not. Statistically significant *p*-values are in bold. a. *p*-values generated using the independent samples *t*-test. b. *p*-value generated using the Mann-Whitney U test.			
**(c) Multiple Sleep Latency Test (MSLT) Features**			
**Variable**	**Anxiety (n = 67)**	**No Anxiety (n = 75)**	***p*-Value**
Avg sleep latency, min median (IQR)	6.20 (5.20–7.40)	4 (2.60–6.40)	**<0.0001 ^a^**
Avg SOREMPs, min mean ± SD (n = 23)	8.83 ± 5.42	9.12 ± 4.40	0.8991 ^b^
Num naps w/ SOREMPS n (%)			**0.0388 ^c^**
0	61 (91.04)	58 (77.33)	
1	6 (8.96)	17 (22.67)	
Abbreviations: MSLT = Multiple Sleep Latency Test, Avg = Average, Min = Minutes, IQR = interquartile range, SOREMPS = Sleep Onset Rapid Eye Movement Periods, SD = Standard Deviation, Num = Number, w/ = With. Notes: Categorical variables are presented as the frequency (n) and percentage (%). Percentages are based upon column totals, unless otherwise noted next to the variable name. Continuous variables are presented as the mean±SD if normally distributed, median and IQR if not. Statistically significant *p*-values are in bold. a. *p*-value generated using the Mann-Whitney U test. b. *p*-value generated using the independent samples *t*-test. c. *p*-value generated using the chi-square test of independence.			

**Table 5:** Clinical and polysomnographic features of patients with and without comorbid dysautonomia.

**(a) Clinical Features**			
**Variable**	**Dysautonomia** **(n = 15)**	**No Dysautonomia** **(n = 127)**	***p*-Value**
IH Symptoms			
Excessive daytime sleepiness	15 (100)	127 (100)	NA
Abruptly falling asleep	0	15 (11.81)	0.3685 ^a^
Cataplexy	0	1 (0.79)	1.0000 ^a^
Sleep paralysis	1 (6.67)	7 (5.51)	1.0000 ^a^
Hypnagogic hallucinations	1 (6.67)	9 (7.09)	1.0000 ^a^
Hypnopompic hallucinations	0	1 (0.79)	1.0000 ^a^
Difficulty falling asleep at night	2 (13.33)	13 (10.24)	0.6604 ^a^
Disrupted sleep	3 (20)	33 (25.98)	0.7607 ^a^
Refreshing naps	2 (13.33)	6 (4.72)	0.2008 ^a^
Non-refreshing naps	8 (53.33)	40 (31.50)	0.0909 ^b^
Sleep inertia	6 (40)	62 (48.82)	0.5179 ^b^
Other	0	1 (0.79)	1.0000 ^a^
ESS at onset (0–24) mean ± SD	16.27 ± 2.91	16.83 ± 2.93	0.4848^c^
ESS after tx or last appt (0–24) mean ± SD	10.47 ± 5.58	11.30 ± 4.55	0.5144 ^c^
Abbreviations: IH = Idiopathic Hypersomnia, ESS = Epworh Sleepiness Scale, and SD = Standard Deviation. Notes: Categorical variables are presented as the frequency (n) and percentage (%). Percentages are based upon column totals, unless otherwise noted next to the variable name. Continuous variables are presented as the mean ± SD if normally distributed or median and IQR if not. Statistically significant *p*-values that are in bold. a. *p*-value generated using Fisher’s exact test. b. *p*-value generated using the chi-square test of independence. c. *p*-value generated using the independent Samples *t*-test.			
**(b) Polysomnography Features**			
**Variable**	**Dysautonomia** **(n = 16)**	**No Dysautonomia** **(n = 140)**	***p*-Value**
Total sleep time, min mean ± SD	397.44 ± 42.83	404.31 ± 31.13	0.4237 ^a^
Total sleep efficiency, % median (IQR)	87 (85–94.50)	90 (86–95)	0.3312 ^b^
Total sleep latency, min median (IQR)	13 (8–28)	13.50 (6–33)	0.7567 ^b^
Total REM latency, min median (IQR)	130 (79.50–173.50)	122.50 (79.50–184.50)	0.8885 ^b^
Abbreviations: min = minutes, SD = Standard Deviation, IQR = Interquartile Range, and REM = Rapid Eye Movement. Notes: Continuous variables are presented as the mean ± SD if normally distributed, median and IQR if not. Statistically significant *p*-values are in bold. a. *p*-values generated using the independent samples *t*-test. b. *p*-value generated using the Mann-Whitney U test.			
**(c) Multiple Sleep Latency Test (MSLT) Features**			
**Variable**	**Dysautonomia** **(n = 15)**	**No Dysautonomia** **(n = 127)**	***p*-Value**
Avg sleep latency, min median (IQR)	7.40 (6–7.80)	5.40 (3–7)	**0.0080 ^a^**
Avg SOREMPs, min mean ± SD (n = 23)	14 (NA)	8.82 ± 4.53	NA
Num naps w/ SOREMPS n (%)			0.4656 ^b^
0	14 (93.33)	105 (82.68)	
1	1 (6.67)	22 (17.32)	
Abbreviations: MSLT = Multiple Sleep Latency Test, Avg = Average, Min = Minutes, IQR = interquartile range, SOREMPS = Sleep Onset Rapid Eye Movement Periods, SD = Standard Deviation, Num = Number, and w/ = With. Notes: Categorical variables are presented as the frequency (n) and percentage (%). Percentages are based upon column totals, unless otherwise noted next to the variable name. Continuous variables are presented as the mean±SD if normally distributed, median and IQR if not. Statistically significant *p*-values are in bold. A *p*-value could not be calculated for average SOREMPs because only 1 patient with dysautonomia had data. a. *p*-value generated using the Mann-Whitney U test. b. *p*-value generated using the Fisher’s exact test.			

## Data Availability

The original contributions presented in this study are included in the article. Further inquiries can be directed to the corresponding author.

## References

[1] Dhillon K., Sankari A. (2025). Idiopathic Hypersomnia. StatPearls.

[2] Morse A.M., Naik S. (2023). Idiopathic Hypersomnia: Neurobiology, Diagnosis, and Management. CNS Drugs.

[3] Arnulf I., Dodet P., Leu-Semenescu S., Maranci J.B. (2023). Idiopathic hypersomnia and Kleine-Levin syndrome. Rev. Neurol..

[4] Dauvilliers Y., Bogan R.K., Arnulf I., Scammell T.E., St Louis E.K., Thorpy M.J. (2022). Clinical considerations for the diagnosis of idiopathic hypersomnia. Sleep Med. Rev..

[5] Sateia M.J. (2014). International classification of sleep disorders-third edition: Highlights and modifications. Chest.

[6] Harris P.A., Taylor R., Thielke R., Payne J., Gonzalez N., Conde J.G. (2009). Research electronic data capture (REDCap)--a metadata-driven methodology and workflow process for providing translational research informatics support. J. Biomed. Inform..

[7] Harris P.A., Taylor R., Minor B.L., Elliott V., Fernandez M., O’Neal L., McLeod L., Delacqua G., Delacqua F., Kirby J. (2019). The REDCap consortium: Building an international community of software platform partners. J. Biomed. Inform..

[8] Krahn L.E., Arand D.L., Avidan A.Y., Davila D.G., DeBassio W.A., Ruoff C.M., Harrod C.G. (2021). Recommended protocols for the Multiple Sleep Latency Test and Maintenance of Wakefulness Test in adults: Guidance from the American Academy of Sleep Medicine. J. Clin. Sleep Med..

[9] Vernet C., Leu-Semenescu S., Buzare M.A., Arnulf I. (2010). Subjective symptoms in idiopathic hypersomnia: Beyond excessive sleepiness. J. Sleep Res..

[10] Vernet C., Arnulf I. (2009). Idiopathic hypersomnia with and without long sleep time: A controlled series of 75 patients. Sleep.

[11] Blattner M., Maski K. (2023). Central Disorders of Hypersomnolence. Continuum.

[12] Blattner M., Maski K. (2023). Narcolepsy and Idiopathic Hypersomnia. Sleep Med. Clin..

[13] Mombelli S., Deshaies-Rugama A.S., Blais H., Sekerovic Z., Thompson C., Desautels A., Montplaisir J., Nigam M., Carrier J., Gosselin N. (2023). Are unrefreshing naps associated with nocturnal sleep architecture specificities in idiopathic hypersomnia?. Sleep.

[14] Plante D.T. (2018). Nocturnal sleep architecture in idiopathic hypersomnia: A systematic review and meta-analysis. Sleep Med..

[15] Murphy M.J., Peterson M.J. (2015). Sleep Disturbances in Depression. Sleep Med. Clin..

[16] Vgontzas A.N., Chrousos G.P. (2002). Sleep, the hypothalamic-pituitary-adrenal axis, and cytokines: Multiple interactions and disturbances in sleep disorders. Endocrinol. Metab. Clin. N. Am..

[17] Barateau L., Lopez R., Franchi J.A., Dauvilliers Y. (2017). Hypersomnolence, Hypersomnia, and Mood Disorders. Curr. Psychiatry Rep..

[18] Plante D.T., Finn L.A., Hagen E.W., Mignot E., Peppard P.E. (2017). Longitudinal associations of hypersomnolence and depression in the Wisconsin Sleep Cohort Study. J. Affect Disord..

[19] Mahmoudi M., Friedman D., Vendrame M., Kothare S.V. (2017). A Case of Recurrent Hypersomnia with Autonomic Dysfunction. J. Clin. Sleep Med..

[20] Adra N., Reddy M., Attarian H., Sahni A.S. (2022). Autonomic dysfunction in idiopathic hypersomnia: An overlooked association and potential management. J. Clin. Sleep Med..

[21] Miglis M.G., Schneider L., Kim P., Cheung J., Trotti L.M. (2020). Frequency and severity of autonomic symptoms in idiopathic hypersomnia. J. Clin. Sleep Med..

